# Prevalence of Hypertension and Associated Factors in Ahvaz School Age Children in 2013

**Published:** 2014-07

**Authors:** Ashrafalsadat Hakim, Roya Bagheri

**Affiliations:** 1 Chronic Disease Care Research Center, Department of Nursing, Ahvaz Jundishapur University of Medical Sciences, Ahvaz, Iran;; 2 Razi Hospital, Ahvaz Jundishapur University of Medical Sciences, Ahvaz, Iran

**Keywords:** Hypertension, Pre Hypertension, Prevalence, School Age Children

## Abstract

**Background:** The underlying cause of high blood pressure in adulthood is rooted in childhood. The evidence points to higher prevalence of hypertension among children in recent years. This study aims to investigate the prevalence of high blood pressure among Ahvaz children, as a sample of Iranian kids, and compare it with relevant reports from other countries.

**Methods:** This cross- sectional study was conducted in various parts of Ahvaz city on school children aged 6-12 from March to June 2013. We measured the height, weight, systolic and diastolic blood pressure, using standardized methods. Systolic and diastolic blood pressure ≥95 percentile for age and sex was identified as hypertension. To analyze the data, statistical tests such as Chi-square, T-test, ANOVA, and Pearson correlation coefficient were used.

**Results:** Overall, 1100 school children (564 boys and 536 girls) participated in the study. The highest level of blood pressure was found among obese children (26.4%) aged 11-12 years. Systolic blood pressure of 9.7% of children was in the pre-hypertension phase and 23.6% of them in the hypertensive phase. Also, with regards to diastolic pressure, 13.5% and 17.1% of the children had pre-hypertension and hypertension, respectively.

**Conclusion:** Considering the high prevalence of hypertension in school children in Ahvaz, we suggest developing a sustainable training program based on intervention for proper nutrition and physical activity in this age group to the Ministry of Health and the Ministry of Education.

## Introduction

Hypertension is one of the leading causes of the global burden of disease. Approximately 7.6 million deaths (13-15% of the total) and 92 million disability-adjusted life years worldwide were attributable to high blood pressure in 2001. Hypertension doubles the risk of cardiovascular diseases, including coronary heart disease (CHD), congestive heart failure (CHF), ischemic and hemorrhagic stroke, renal failure, and peripheral arterial disease.^1^

Elevated blood pressure in childhood is a significant risk factor for cardiovascular disease in adulthood.^2^ “In industrialized societies, blood pressure (BP) increases steadily during the first two decades of life. In children and adolescents, BP is associated with growth and maturation”.^1^ BP changes depending on age and growth, and as the person gets older the heart enlarges and the likelihood of blood pressure increases.^3^ The evidence shows that a minor increase in blood pressure among children and adolescents is much more common than already thought. High blood pressure in adulthood can be traced back to childhood; also, hypertension in childhood continues to adulthood. In fact, it is said that children with high blood pressure are more likely to be at risk of developing high blood pressure later in life.^4^ In children and adolescents, hypertension usually is defined as systolic and diastolic blood pressure which is constantly over the 95th percentile for age, sex and height. In addition blood pressure between 90-95 percentiles is considered as pre- hypertension.^1^^,^^5^

Both genetic and environmental factors as well as regional and racial variations are likely to have an impact on the prevalence of hypertension. Obesity and overweight are predominant risk factors for High BP. Adaptation of lifestyles that favorably affect blood pressure has implications for both prevention and treatment of hypertension. Health-promoting lifestyle modifications are recommended for individuals with pre-hypertension and as an adjunct to drug therapy in hypertensive individuals.^1^

Lifestyle changes that effectively lower the blood pressure are weight loss, regular physical activity, reduced salt intake, increased potassium intake and an overall healthy dietary pattern.^1^ Several studies in Iran have been conducted on high BP in children. For example, a study showed that systolic BP of 11.6% of children aged 6-11 in Birjand were pre-hypertensive and 7.4% were hypertensive.^6^ In the same line, another study indicated that prevalence of high BP in girls ranged between 4.4 to 9.6 percent and  it was between 3 to 8.2 percent for boys.^7^

Thus, as the motto of 1392 goes “take blood pressure seriously” and also due to implications of high blood pressure in adulthood dating back to childhood, the researcher aimed to investigate the prevalence of high blood pressure among school-age children in Ahvaz, 2013.

## Patients and Methods

This cross-sectional survey was performed in four educational regions in Ahvaz city on 1100 male and female students. Of them, 536 were girls and the other 564 boys. The sampling was done during March until June 2013.  The sample size was calculated according to previous related articles and using the following formula.

n=N^2^Z pq/d^2^(N-1)+z^2^ pq

N=the whole population of Ahvaz children in Schools=137993

Z=1.96

p=the blood pressure ratio in articles=0.5

q=1-p=0.5

d=0.03

The figure 1060 was drawn out of the above formula, but 1100 was chosen presuming some drop outs. The method used in this research is cluster sampling. Thus two schools from each region were selected randomly (one girls’ school and the other boys’ .Subsequently, about 22 students were selected from each grade (1^th^- 6^th^ primary school grades), using random sampling according to the number of students. After determining the number of samples and their choices, written consent was obtained from parents, school officials, and the deputy.

The height, weight and blood pressure were measured by the researcher. Data collection procedure was carried out between 8 to 12 am in their respective schools and health rooms where first the height then weight and finally the blood pressure were measured. The student demographic questionnaire was filled out for each participant. The criterion for age calculation was their actual age in years (adjusted with ID); it was then matched with their school year. Height was measured by a tape and in centimeters. Weight was measured in kg using seca scale made in Germany with 0.01 gram readability. In all cases, careful balance of the scale was maintained. Children’s BP was measured after a ten minute break from the right hand in a sedentary state using analog barometer alpk II (made ​​in Japan). Blood pressure measurement was performed by two types of cuffs (depending on the student’s arm size) in sizes 8×18 and 9×20 cm.  Measurement of blood pressure was done on two separate points in time with 20 minute interval.^5^ The individual’s mean BP was considered. Body mass index (BMI) for each sample was calculated as the ratio of weight in kg by the square of height in m^2^. To determine overweight and obesity, percentiles provided by the center for disease control and prevention was used.^8^ So 85 to 95th percentile for age and sex was defined as overweight and obesity as 95 percentile and higher.

The criterion for high blood pressure was in accordance with the data presented in the tables of National Training Center program for high blood pressure in 2012. These tables represent the systolic and diastolic blood pressure based on age, sex and height. The percentile 90 to 95 was considered as pre-hypertension and the that equal or above 95 as hypertension.^5^^,^^9^

Samples were selected from healthy students of each school and children with a history of cardiovascular, renal and endocrine diseases were excluded from the study. Data were collected, using SPSS software, version 20, and for the analysis of the data, K2, T-test, ANOVA, Pearson Correlation Coefficient tests were used.

## Results

1100 students participated in this study. Most of them were in the age range of 11-12 years (19.2%). 536 (48.7%) students were girls and 564 (51.3%) boys. The systolic blood pressure of 9.7% of the children was in the pre-hypertensive stage and 23.8% of them were hypertensive. Also in regards with the diastolic pressure, 13.5% and 17.1% of the children were pre-hypertensive and hypertensive, respectively (table 1).

**Table 1:** Distribution of children’s blood pressure in terms of systolic and diastolic blood pressure

**Variable**	**Systolic BP**		**Diastolic BP**	
	**Percent**	**Number**	**Percent**	**Number**
Normal BP	66.6	733	69.5	764
Pre-hypertensive	9.7	107	13.5	148
Hypertensive	23.6	260	17.1	188
Total	100	1100	100	1100

Due to normality of data distribution based on independent *t* test, it was revealed that the mean systolic blood pressure in girls was significantly higher than boys. But in terms of mean diastolic pressure there was no statistically significant difference between male and female students and similar BP was observed in both groups (table 2).

**Table 2:** Comparison between mean and standard deviation of systolic and diastolic blood pressure in students based on sex

**Characteristics**	**Mean**		**Standard deviation**		**Level of** **Significance**
	**Girls**	**Boys**	**Girls**	**Boys**	
Number	536	564	536	564	
Systolic BP	111.13	109.34	17.26	12.54	0.048
Diastolic BP	68.38	68.78	10.67	9.39	0.508

Distribution in Table 3 shows children’s systolic and diastolic blood pressure according to sex. Findings indicated that girls had a higher systolic blood pressure (28.5%) than boys (18.8%). Also, diastolic blood presser in girls (19%) was higher than boys (15.2%). But boys had higher pre-hypertensive systolic and diastolic BP than girls (table 3).

**Table 3:** Distribution of children’s systolic and diastolic BP based on sex

**Sex**	**Variable**	**Systolic BP**		**Diastolic BP**	
		**Percent**	**Number**	**Percent**	**Number**
Girls	Normal BP	64	343	68.7	368
	Pre-hypertensive	7.5	40	12.3	66
	Hypertensive	28.5	153	19	102
	Total	100	536	100	536
Boys	Normal BP	69.1	390	70.2	396
	Pre-hypertensive	11.9	67	14.5	82
	Hypertensive	18.8	106	15.2	86
	Total	100	564	100	564

According to Turkey’s post-hoc test, it was determined that mean systolic BP between age groups of 6-7, 7-8, 8-9 years old and age groups of 9-10, 10-11, 11-12 was statistically significant and the age group of 9 to 12 had a higher mean systolic BP.

Also it was determined that the mean diastolic BP between the age group of 6-7 and age group of 9 to 12 had a statistically significant difference; also, there was a significant difference between the age groups of 7-8 and 8-9 years with the age group of 11-12.  This indicates that those in the age group of 11-12 years old have higher diastolic blood pressure (table 4).

**Table 4:** Turkey’s systolic and diastolic post-hoc test based on age

**Age**	**Number**	**Mean systolic BP**	**Standard error**	**Mean diastolic BP**	**Standard error**
6-7	197	106.82	1.38	65.42	0.92
7-8	250	107.68	1.66	67.71	1.10
8-9	142	108.30	1.61	67.85	1.07
9-10	140	112.36	1.51	69.93	1.01
10-11	182	111.78	1.49	69.37	1.00
11-12	189	115.52	1.53	71.89	1.02

Children were classified into three groups of obese, overweight and normal weight according to their body max index.

Given the calculated significant level (P<0.05) the prevalence of hypertension increased significantly in accordance with increased BMI (table 5).

**Table 5:** Prevalence of hypertension in accordance with BMI

**Characteristics**	**Normal weight** **(mean±SD)**	**Overweight** **(mean±SD)**	**Obese** **(mean±SD)**	**P value**
Normal BP	655 (90.8)	128 (84.2)	167 (73.6)	0.001
Elevated BP	66 (9.2)	24 (15.8)	60 (26.4)	

## Discussion

In the present study, the prevalence of hypertension in children aged 6-12 in Ahvaz city was 23.6% and that of pre-hypertension was 9.7%. But in other study results the prevalence rates of hypertension and pre-hypertension were 7.4% and 11.5%, respectively;  also other study results have indicated 11.8 and 15.2.^6^^,^^10^ As mentioned before, the prevalence of hypertension in this study is far higher than others studies,^6^^,^^10^ but that of pre-hypertension is much less which indicates that the number of children with hypertension in Ahvaz has increased. It seems that the above results point out to differences in the type and amount of nutrition and perhaps lack of physical activity.

In the same line, other studies have reported a much lower prevalence of hypertension in school-age children. In a study in Zahedan  conducted on 1500 primary school children, the systolic BP was comparable with international standards.^11^ Also, the results of another study  on 1061 primary school children in Tehran showed that Tehrani kids had lower systolic and diastolic BP than normal blood pressure percentile in American kids.^12^ Since the above studies were conducted a decade ago, it seems that an increase in the prevalence of hypertension in kids is related to the increasing pace of industrialization of societies and sedentary lifestyle and overweight and obesity epidemic.

In the current study, systolic BP among female students was significantly higher than male students; this is inconsistent with the result of another study.^6^ But it is in the same line with the results from  another study.^12^ The reason for this difference could be the impact of the geographic zone, earlier puberty in girls than boys, and as a result the elevated BP in girls due to puberty hormones.

This study showed that the mean systolic and diastolic blood pressure increases with age so that the age group of 11-12 had higher systolic and diastolic BP than younger age groups; this is consistent with the findings of another study results.^13^ It appears that the increase in cardiac mass with aging results in the increase of blood pressure levels in older age groups.

In this study, hypertension in overweight and obese students was significantly higher than those with normal weight. The same results were found in another study.^14^ A study conducted between 2000 and 2010 in China revealed that the prevalence of hypertension from 2000 through 2010 rose from 19.29% to 26.16% in boys and from 14.69% to 19.77% in girls. Also the prevalence of obesity rose from 22.26% to 32.81% in boys and from 12.22% to 19.49% in girls. These results also corroborate with those of a study in Texas, USA.^15^ Probably, the epidemic of child obesity and a strong correlation between blood pressure and body weight have increased the prevalence of hypertension in the young population. The roots of obesity as a major cause of hypertension in childhood can lie in the consumption of high-calorie foods such as fast food and carbonated beverages and reduced physical activity due to the replacement of outdoor and active games with watching TV and PC games.

## Conclusion

According to the findings of this Study, it seems that prevalence of hypertension in Ahvaz school children is high. In this survey, female sex, overweight, obesity and older age were observed as risk factors for high blood pressure among the students. Some essential approaches for primary prevention can be suggested, such as written educational programs for proper nutrition, physical activity and establishing healthy recreations. Furthermore, follow up examinations of blood pressure in six months and one year intervals in children exposed to risk or suffering from high BP is recommended. Hence holding an intervention program by Ministry of Health and Ministry of Education in the interest of prevention and treatment of high blood pressure is suggested. 
